# The Receptor for Activated C Kinase in Plant Signaling: Tale of a Promiscuous Little Molecule

**DOI:** 10.3389/fpls.2015.01090

**Published:** 2015-12-08

**Authors:** Tania Islas-Flores, Ahasanur Rahman, Hemayet Ullah, Marco A. Villanueva

**Affiliations:** ^1^Unidad Académica de Sistemas Arrecifales, Instituto de Ciencias del Mar y Limnología, Universidad Nacional Autónoma de MéxicoPuerto Morelos, México; ^2^Department of Biology, Howard UniversityWashington, DC, USA

**Keywords:** hormonal regulation, RACK1, signal transduction, WD-repeat protein, WD40 domain

## Abstract

Two decades after the first report of the plant homolog of the Receptor for Activated C Kinase 1 (RACK1) in cultured tobacco BY2 cells, a significant advancement has been made in the elucidation of its cellular and molecular role. The protein is now implicated in many biological functions including protein translation, multiple hormonal responses, developmental processes, pathogen infection resistance, environmental stress responses, and miRNA production. Such multiple functional roles are consistent with the scaffolding nature of the plant RACK1 protein. A significant advance was achieved when the β-propeller structure of the *Arabidopsis* RACK1A isoform was elucidated, thus revealing that its conserved seven WD repeats also assembled into this typical topology. From its crystal structure, it became apparent that it shares the structural platform for the interaction with ligands identified in other systems such as mammals. Although RACK1 proteins maintain conserved Protein Kinase C binding sites, the lack of a *bona fide* PKC adds complexity and enigma to the nature of the ligand partners with which RACK1 interacts in plants. Nevertheless, ligands recently identified using the split-ubiquitin based and conventional yeast two-hybrid assays, have revealed that plant RACK1 is involved in several processes that include defense response, drought and salt stress, ribosomal function, cell wall biogenesis, and photosynthesis. The information acquired indicates that, in spite of the high degree of conservation of its structure, the functions of the plant RACK1 homolog appear to be distinct and diverse from those in yeast, mammals, insects, etc. In this review, we take a critical look at the novel information regarding the many functions in which plant RACK1 has been reported to participate, with a special emphasis on the information on its currently identified and missing ligand partners.

## Introduction

Receptors for activated C kinase (RACKs) were initially described as ~33 kDa proteins from particulate fractions of rat heart, which fulfilled the criteria of intracellular receptors for the activated enzyme (Mochly-Rosen et al., 1991a,b). The first cloned sequence encoding one of such receptors was obtained from a rat brain cDNA expression library and termed RACK1 due to its ability to bind activated protein kinase C (PKC) (Ron et al., 1994). RACK1 is now known to be a highly conserved intracellular adaptor protein belonging to the WD-repeat family only present in eukaryotic organisms. Its constitutive expression and ubiquitous presence in both unicellular and multicellular organisms highlights its functional importance. The protein sequence contains seven WD-40 domains which assemble into a typical seven-bladed β-propeller structure. Since this assembly provides an interactive platform for the binding of potential ligand partners in proximity, the notion that RACK1 only functions as a receptor for active PKC isoforms has changed to that of a versatile protein that also provides a scaffold for direct or indirect interaction with many different ligands. This ability has positioned RACK1 as a central hub for integration of multiple pathways that impinge key cellular functions (Reviewed in Adams et al., 2011).

Much of our current knowledge about RACK1 function has arisen from mammalian cell studies; however, new emerging models such as yeast, slime molds, and worms have also provided clues to the variety of biochemical pathways in which RACK1 participates. For example, the *Saccharomyces cerevisiae* homolog Asc1 has been reported to regulate protein translation critical for cell wall integrity near the yeast budding sites (Melamed et al., 2010). DdRACK1 from *Dyctiostelium discoideum*, has been reported to interact with heterotrimeric G proteins and impact growth and developmental processes (Omosigho et al., 2014). Finally, the *Caenorhabditis elegans*, RACK1 homolog interacts with the actin-binding protein UNC-115/abLIM for axon pathfinding and lamellipodia and filopodia formation, while in an independent and novel signaling pathway, it participates in gonadal tip cell migration (Demarco and Lundquist, 2010).

**Figure 1:**
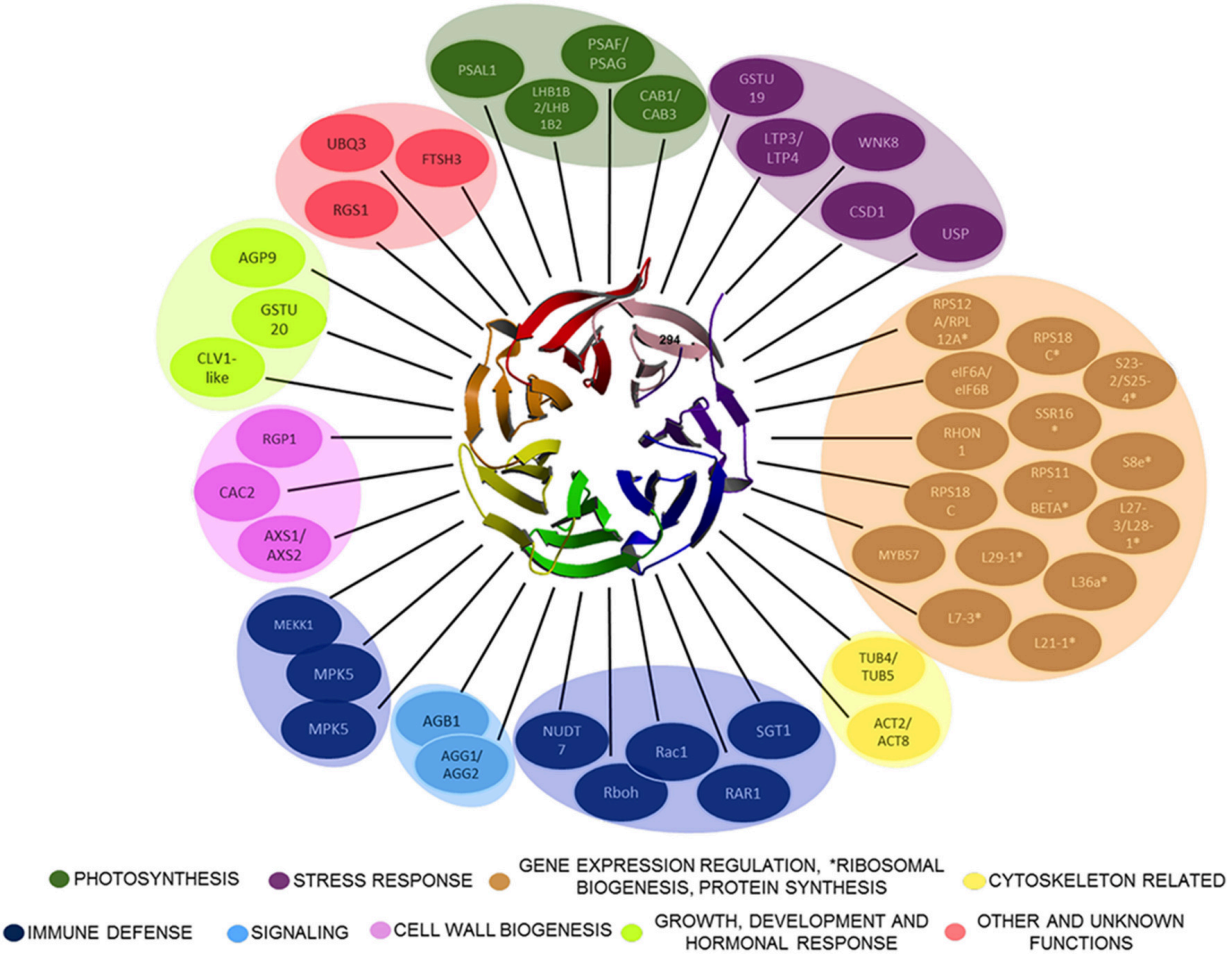
**Plant RACK1 is related with several cellular processes through its interacting ligands**. Only some of the proteins demonstrated to interact directly with RACK1 are outlined. The β-propeller structure at the center corresponds to the top view of AtRACK1 (Ullah et al., 2008). The RACK1 interactors belong to different cellular processes ranging from Cell wall biogenesis, Photosynthesis, Cytoskeletal, Immune defense, Stress response, Signaling, Gene expression regulation, ^*^Ribosomal biogenesis, Protein synthesis, Growth, Development and hormonal response, to Other and unknown.

Besides the many model organisms in which RACK1 has been studied, plant RACK1 homologs have also been described. The first report was that of the auxin-regulated RACK1 homolog arcA (*ArcA* for auxin-regulated gene from cultured cells) from tobacco BY2 cells (Ishida et al., 1993). Since then, plant RACK1 homologs were reported from *Oryza sativa* (Iwasaki et al., 1995; Nakashima et al., 2008), *Brassica napus* (Kwak et al., 1997), *Medicago sativa* (McKhann et al., 1997), *Solanum lycopersicum* (Kiyosue and Ryan, 1999), *Glycine max* (Nielsen et al., 2001); *Arabidopsis thaliana* (van Nocker and Ludwig, 2003), *Phaseolus vulgaris* (Islas-Flores et al., 2009), and *Zea mays* (Wang et al., 2014), among others. In animal cells, a single copy of *RACK1* is encoded; however, this number is variable in plant genomes. For example, the genome of *A. thaliana* encodes three *RACK1* genes termed *AtRACK1A* (At1g18080), *AtRACK1B* (At1g48630), and *AtRACK1C* (At1g18130) (Chen et al., 2006), while two genes are present in *O*. *sativa* (Zhang et al., 2014). To date, the evidence acquired indicates that plant RACK1 does not have a unique function but rather, it is emerging as a multi-functional protein playing a central role in critical biological responses (Figure 1). Through cryo-electron microscopy (Cryo-EM) studies using ribosomes from the fungi *Thermomyces lanuginosus* and *Saccharomyces cereviseae*, it was revealed that RACK1 is a component of the ribosomal 40S subunit (Sengupta et al., 2004) that anchors PKC, a family of serine/threonine kinases, to phosphorylate the eukaryotic Initiation Factor6 (eIF6). In addition to binding with PKC, RACK1 plays crucial role in protein translation, tissue development, circadian clock, neural responses and tumorigenesis in mammals (McCahill et al., 2002; Robles et al., 2010). In plants, RACK1 regulates various signaling pathways ranging from developmental processes such as seed germination, flowering and leaf production, to immune and stress responses against pathogen and environmental stimuli (Chen et al., 2006; Ullah et al., 2006). Intriguing however, despite the recent findings of new plant RACK1 ligands, has been the search for signal-transduction and/or biochemical pathways in which plant RACK1 is involved. In this review, we cover and critically discuss past and present findings regarding the various signaling and biochemical pathways in which plant RACK1 has been reported to participate. In addition, we take a critical look at the various binding partners with which it has been reported to interact, and discuss the present trends and directions of the field.

## WD-repeat protein RACK1: a small inbuilt β-propeller molecule with large responsibilities

WD-40 domains assemble into β sheets to form blade-like structures, and proteins containing seven WD-40 repeats such as the β subunit of the heterotrimeric G protein can assemble into a seven-bladed β-propeller-like structure (Reviewed in McCahill et al., 2002). The β-propeller is a molecular platform that facilitates interaction with several ligands from distinct signaling pathways at the same time. RACK1 is a 36 kDa cytosolic protein with high sequence identity with the β subunit of heterotrimeric G proteins, and highly conserved in eukaryotes where it shares 43–76% of sequence identity (Ullah et al., 2008). Consequently, the WD-40 repeats are highly conserved in all RACK1 homologs characterized so far, indicating that all of them share the same structure. This assumption was supported by the first crystallographic resolution of the AtRACK1A isoform structure at 2.4 Å (Ullah et al., 2008). Subsequent determinations of the crystallographic structure for RACK1 from different organisms, such as, *S. cerevisiae* (Yatime et al., 2011), *Tetrahymena thermophila* (Rabl et al., 2011), and *Homo sapiens* (Ruiz Carrillo et al., 2012) at 2.1, 2.4, 3.9, and 2.45 Å resolution, also confirmed the β-propeller structure platform. Thus, the available crystal structures have shown that its seven-bladed structural dynamics presents the features to scaffold a large number of proteins to facilitate numerous cellular signaling pathways. As of now, more than 130 different interacting partners of plant RACK1 homologs have been reported (Table 1 and Figure 1), suggesting its involvement in various physiological functions in this kingdom (Ullah et al., 2006; Kundu et al., 2013).

**Table 1:** **Plant RACK1 ligand proteins**.

	**RACK1 isoform**	**Ligand ID**	**Name of the ligand**	**Process**	**Assay**	**Specie**	**References**
**CELL WALL BIOGENESIS**							
1	AtRACK1C	AT1G08200	AXS2 (UDP-D-APIOSE/UDP-D-XYLOSE SYNTHASE 2); UDP-glucuronate decarboxylase	Cell wall organization	c	*A. thaliana*	Klopffleisch et al., 2011
2	AtRACK1A	AT1G20220	Alba DNA/RNA binding protein (NP 564108.1)	Cell wall related	e	*A. thaliana*	Kundu et al., 2013
3	AtRACK1A/AtRACK1C	AT2G27860	AXS1 (UDP-D-apiose/UDP-D-xylose synthase 1); NAD or NADH Binding/UDP-glucuronate decarboxylase	Cell wall organization	c, g	*A. thaliana*	Klopffleisch et al., 2011
4	AtRACK1C	AT3G02230	RGP1, Reversibly glycosylated polypeptide 1; UDP-arabinose mutase 1	Cell wall biogenesis	c	*A. thaliana*	Klopffleisch et al., 2011
5	AtRACK1C	AT3G25150	NTF2, Nuclear transport factor 2 family; protein with RNA Binding (RRM-RBD-RNP motifs) domain	Cell wall organization	c	*A. thaliana*	Klopffleisch et al., 2011
6	AtRACK1C	AT5G14430	PMT9, S-adenosyl-L-methionine-dependent methyltransferases superfamily protein	Cell wall related	c	*A. thaliana*	Klopffleisch et al., 2011
7	AtRACK1A	AT5G35360	CAC2, Biotin carboxylase subunit	Cell wall organization/Fatty acid biosynthesis	e	*A. thaliana*	Kundu et al., 2013
**PHOTOSYNTHESIS**							
8	AtRACK1A	AT1G20340	Plastocyanin major isoform, DNA-damage resistance protein (DRT112)	Light response	e, f	*A. thaliana*	Kundu et al., 2013
9	AtRACK1A	AT1G29910	CAB3, Chlorophyll A/B binding protein 3	Photosynthesis	e	*A. thaliana*	Kundu et al., 2013
10	AtRACK1A	AT1G29930	CAB1, Chlorophyll A/B binding protein1	Photosynthesis	e, f	*A. thaliana*	Kundu et al., 2013
11	AtRACK1A	AT1G31330	PSAF, Photosystem I subunit F	Photosynthesis	e	*A. thaliana*	Kundu et al., 2013
12	AtRACK1A	AT1G55670	PSAG, Photosystem I subunit G	Electron transport in photosystem I	e	*A. thaliana*	Kundu et al., 2013
13	AtRACK1A	AT1G59840	CCB4, Cofactor assembly of complex C	Photosystem II assembly	e	*A. thaliana*	Kundu et al., 2013
14	AtRACK1A	AT1G60950	FED A, Ferredoxin-2	Light response	e	*A. thaliana*	Kundu et al., 2013
15	AtRACK1A	AT1G61520	LHCA3, Photosystem I Light Harvesting Complex Gene 3	Light harvesting	e, f	*A. thaliana*	Kundu et al., 2013
16	AtRACK1A	AT1G67090	RuBisCO1A, Rubisco small subunit 1A	Light response/Photosyntesis	e, f	*A. thaliana*	Kundu et al., 2013
17	AtRACK1A	AT2G34420	LHB1B2, Photosystem II light harvesting complex protein B1B2	Light harvesting	e	*A. thaliana*	Kundu et al., 2013
18	AtRACK1A	AT2G34430	LHB1B1, Light-harvesting chlorophyll protein complex II subunit B1	Light harvesting	e	*A. thaliana*	Kundu et al., 2013
19	AtRACK1A	AT2G43560	FKBP16-3, Peptidyl-prolyl cis-trans isomerase	Protein folding/Photosynthesis	e	*A. thaliana*	Kundu et al., 2013
20	AtRACK1A	AT3G56940	CRD1, Magnesium-protoporphyrin IX monomethyl ester [oxidative] cyclase	Chlorophyll biosynthesis	e	*A. thaliana*	Kundu et al., 2013
21	AtRACK1A	AT3G61470	LHCA2, Photosystem I Light Harvesting Complex Gene 2	Light harvesting	e, f	*A. thaliana*	Kundu et al., 2013
22	AtRACK1A	AT3G62410	CP12-2, Domain-containing protein 1	Light response	e	*A. thaliana*	Kundu et al., 2013
23	AtRACK1A	AT4G12800	PSAL, Photosystem I subunit L; Photosystem I reaction center subunit XI	Light reaction/Photosystem II assembly	e	*A. thaliana*	Kundu et al., 2013
24	AtRACK1A	AT5G38430	RuBisCO1B, Small chain 1B	Light response	e	*A. thaliana*	Kundu et al., 2013
25	AtRACK1A	AT5G64040	PSAN, Photosystem I reaction center subunit N	Electron transport in photosystem I	e	*A. thaliana*	Kundu et al., 2013
**CYTOSKELETON RELATED**							
26	AtRACK1A	AT1G20010	TUB5, Tubulin beta-5 chain	Cytoskeleton organization	e	*A. thaliana*	Kundu et al., 2013
27	AtRACK1A	AT1G49240	ACT8, Actin 8	Cytoskeleton organization	e	*A. thaliana*	Kundu et al., 2013
28	AtRACK1A	AT3G18780	ACT2, Actin 2	Cytoskeleton organization	e	*A. thaliana*	Kundu et al., 2013
29	AtRACK1A	AT4G20890	TUB9, Tubulin beta-9 chain	Cytoskeleton organization	e	*A. thaliana*	Kundu et al., 2013
30	AtRACK1A	AT5G23860	TUB8, Tubulin beta-8 chain	Cytoskeleton organization	e	*A. thaliana*	Kundu et al., 2013
31	AtRACK1A	AT5G44340	TUB4, Tubulin beta-4 chain	Cytoskeleton organization	e	*A. thaliana*	Kundu et al., 2013
**IMMUNE DEFENSE**							
32	OsRACK1A	AAF18438	SGT1, Suppressor of G2 allele of Skp1	Disease resistance	b, d, f	*O. sativa*	Nakashima et al., 2008
33	OsRACK1A/OsRACK1B	AB029508	Rac1, Small GTP-binding protein 1	Disease resistance/cell death	a, b, d, f	*O. sativa*	Nakashima et al., 2008
34	OsRACK1A	AB029510	Rac3, Small GTP-binding protein 3; Rho family small GTPases	Disease resistance/cell death	f	*O. sativa*	Nakashima et al., 2008
35	OsRACK1A	AK058414	Rac7, Small GTP-binding protein 7; Rho family small GTPases	Disease resistance/cell death	f	*O. sativa*	Nakashima et al., 2008
36	OsRACK1A	AK067504	Rac5, Small GTP-binding protein 5; Rho family small GTPases	Disease resistance/cell death	f	*O. sativa*	Nakashima et al., 2008
37	OsRACK1A	AK100842	Rac6, Small GTP-binding protein 7; Rho family small GTPases	Disease resistance/cell death	f	*O. sativa*	Nakashima et al., 2008
38	OsRACK1A	AK111881	RAR1, Required for *Mla12* resistance; zinc-binding protein	Disease resistance	b, d, f	*O. sativa*	Nakashima et al., 2008
39	AtRACK1A	AT1G07790	H2B Histone	Defense response	e	*A. thaliana*	Kundu et al., 2013
40	AtRACK1A	AT2G16870	TIR-NBS-LRR class disease resistance protein	Defense response	e	*A. thaliana*	Kundu et al., 2013
41	AtRACK1A	AT2G43790	MPK6, MAP kinase 6	Defense response	b, g, h	*A. thaliana*	Cheng et al., 2015
42	AtRACK1A	AT3G01500	CA1, Carbonic anhydrase 1	Defense response	e, f	*A. thaliana*	Kundu et al., 2013
43	AtRACK1A	AT3G12780	PGK1, Phosphoglycerate kinase 1	Defense and stress response	e	*A. thaliana*	Kundu et al., 2013
44	AtRACK1A	AT3G21220	MKK5, Mitogen-activated protein kinase kinase 5	Defense response	b, g, h	*A. thaliana*	Cheng et al., 2015
45	AtRACK1A	AT3G45640	MPK3, Mitogen-activated protein kinase 3	Defense response	b, g, h	*A. thaliana*	Cheng et al., 2015
46	AtRACK1A	AT3G62030	ROC4, Peptidyl-prolyl cis-trans isomerase CYP20-3	Defense response	e, f	*A. thaliana*	Kundu et al., 2013
47	AtRACK1A	AT4G00870	Transcription factor bHLH14	Defense response	e	*A. thaliana*	Kundu et al., 2013
48	AtRACK1A/AtRACK1b/AtRACK1C	AT4G08500	MEKK1, Mitogen-activated protein kinase kinase kinase 1 in a inactive form (K361M)	Stress and defense response	b, g, h	*A. thaliana*	Cheng et al., 2015
49	AtRACK1A	AT4G12720	NUDT7, Nudix hydrolase 7	Defense and oxidative stress response	b, d, g	*A. thaliana*	Olejnik et al., 2011
50	AtRACK1A	AT5G04140	GLU1 Ferredoxin-dependent glutamate synthase 1 (Fd-GOGaT)	Defense and stress response	e	*A. thaliana*	Kundu et al., 2013
51	AtRACK1C	AT5G09650	PPa6, Soluble inorganic pyrophosphatase 1	Defense and stress response	c	*A. thaliana*	Klopffleisch et al., 2011
52	AtRACK1A	AT5G20630	GER3, Germin-like protein subfamily 3 member 3	Defense and stress response	e	*A. thaliana*	Kundu et al., 2013
53	AtRACK1A	AT5G24780	VSP1, Vegetative storage protein 1 acid phosphatase	Defense response	e	*A. thaliana*	Kundu et al., 2013
54	OsRACK1A	AY603975	Rboh, NADPH oxidase	Defense response	f	*O. sativa*	Nakashima et al., 2008
**STRESS RESPONSE**							
55	AtRACK1A	AT1G08830	CSD1, Superoxide dismutase [cu-Zn]	Response to oxidative stress	e, f	*A. thaliana*	Kundu et al., 2013
56	AtRACK1A	AT1G32640	MYC2 Transcription factor	Response to drought stress and ABA treatment	e	*A. thaliana*	Kundu et al., 2013
57	AtRACK1A	AT1G52420	UDP-Glycosyltransferase-like protein	Response to hypoxia	e	*A. thaliana*	Kundu et al., 2013
58	AtRACK1A/AtRACK1C	AT1G78380	GSTU19, TAU 19 Glutathione S-transferase	Response to drought and oxidative stress	c, e	*A. thaliana*	Kundu et al., 2013; Klopffleisch et al., 2011
59	AtRACK1A/AtRACK1C	AT2G01140	Fructose-bisphosphate aldolase 3	Response to oxidative and saline stress	c, e	*A. thaliana*	Kundu et al., 2013; Klopffleisch et al., 2011
60	AtRACK1A	AT3G52300	ATPQ, ATP synthase subunit D	Response to saline stress	e	*A. thaliana*	Kundu et al., 2013
61	AtRACK1A/AtRACK1C	AT3G53990	USP, Universal stress protein family protein/Adenine nucleotide alpha hydrolases-like superfamily protein	Cold stress response	c, g	*A. thaliana*	Klopffleisch et al., 2011
62	AtRACK1A	AT5G20010	RAN-1 GTP-binding nuclear protein Ran-1; RAS-related nuclear protein-1	Response to saline stress/Protein import into the nucleus	e	*A. thaliana*	Kundu et al., 2013
63	AtRACK1C	AT5G20150	SPX1 domain-containing protein 1	Response to stress starvation	c, g	*A. thaliana*	Klopffleisch et al., 2011
64	AtRACK1A/AtRACK1B/AtRACK1C	AT5G41990	WNK8, WITH NO LYSINE (K) KINASE 8; Serine/threonine-protein kinase	Response to saline and osmotic stress/ Response to glucose	c, d, g	*A. thaliana*	Klopffleisch et al., 2011; Urano et al., 2015
65	AtRACK1A	AT5G59310	LTP4, Non-specific lipid-transfer protein 4	Response to drought stress	e	*A. thaliana*	Kundu et al., 2013
66	AtRACK1A	AT5G59320	LTP3, Non-specific lipid transfer protein 3	Response to drought stress	e	*A. thaliana*	Kundu et al., 2013
**SIGNALING**							
67	AtRACK1A	AT1G05000	Tyrosine phosphatase family; Atypical dual specificity phosphatase 1	Signaling	c, d	*A. thaliana*	Klopffleisch et al., 2011
68	AtRACK1B	AT3G18130	AtRACK1C, Receptor for Activated C Kinase 1C	Signaling	c, g	*A. thaliana*	Klopffleisch et al., 2011
69	AtRACK1A	AT3G22942	AGG2, G-protein gamma-subunit 2	Signaling	b, d, g	*A. thaliana*	Olejnik et al., 2011
70	AtRACK1A	AT3G63420	AGG1, G-protein gamma-subunit 1	Signaling	b, d, g	*A. thaliana*	Olejnik et al., 2011
71	AtRACK1A/AtRACK1B/AtRACK1C	AT4G34460	AGB1, G-protein beta-subunit 1	Signaling	b, g, h	*A. thaliana*	Cheng et al., 2015
72	AtRACK1A	AT4G34870	ROC5, Rotamase cyclophilin 5; Peptidyl-prolyl cis-trans isomerase CYP18-4	Signal transduction	e	*A. thaliana*	Kundu et al., 2013
**GENE EXPRESSION REGULATION**							
73	AtRACK1A	AT1G06190	RHON1, Rho termination factor, N-terminal domain; Ribonucleic acid-binding protein	Transcription termination	e	*A. thaliana*	Kundu et al., 2013
74	AtRACK1A	AT1G09250	bHLH149, Basic helix-loop-helix DNA-binding superfamily protein	Transcription	e	*A. thaliana*	Kundu et al., 2013
75	AtRACK1A	AT1G09590	L21-1, 60S Ribosomal protein	Translation	e	*A. thaliana*	Kundu et al., 2013
76	AtRACK1A	AT1G15930	RPS12A, 40S Ribosomal protein S12-1	Translation/Stress response	e	*A. thaliana*	Kundu et al., 2013
77	AtRACK1A	AT2G18090	PHD Finger, SWIB/MDM2 and GYF domain-containing protein	Transcription	e	*A. thaliana*	Kundu et al., 2013
78	AtRACK1A	AT2G19730	L28-1, 60S Ribosomal protein	Translation	e	*A. thaliana*	Kundu et al., 2013
79	AtRACK1A/AtRACK1B/AtRACK1C	AT2G27100	SE, Serrate RNA effector molecule; miRNA factor	Pri-miRNA processing	b, c, d, g	*A. thaliana*	Speth et al., 2013
80	AtRACK1A/AtRACK1B/AtRACK1C	AT2G39820	eIF6B, 60S Ribosomal protein	Translation/Ribosome biogenesis	d, g	*A. thaliana*	Guo et al., 2011b
81	AtRACK1A	AT2G44120	L7-3, 60S Ribosomal protein	Translation	e	*A. thaliana*	Kundu et al., 2013
82	AtRACK1A	AT3G01530	MYB57, Myb domain protein 57	Transcription	e	*A. thaliana*	Kundu et al., 2013
83	AtRACK1A	AT3G06700	L29-1, 60S Ribosomal protein	Translation/Ribosome biogenesis	e	*A. thaliana*	Kundu et al., 2013
84	AtRACK1A	AT3G23390	L36a, 60S Ribosomal protein	Translation	e	*A. thaliana*	Kundu et al., 2013
85	AtRACK1A	AT3G27830	RPL12-A, 50S Ribosomal protein L12-1	Translation	e	*A. thaliana*	Kundu et al., 2013
86	AtRACK1A/AtRACK1B/AtRACK1C	AT3G55620	eIF6A, 60S Ribosomal protein	Translation/Ribosome biogenesis	d, g	*A. thaliana*	Guo et al., 2011b
87	AtRACK1A	AT4G09800	RPS18C, 40S Ribosomal protein S18	Translation	e	*A. thaliana*	Kundu et al., 2013
88	AtRACK1C	AT4G13940	HOG1, Homology-dependent gene silencing 1; MEE58 adenosylhomocysteinase 1; SAHH1, EMB1395, SAH1	Post-transcriptional gene silencing	c	*A. thaliana*	Klopffleisch et al., 2011
89	AtRACK1A	AT4G15000	L27-3, 60S Ribosomal protein	Translation	e	*A. thaliana*	Kundu et al., 2013
90	AtRACK1A	AT4G21660	Splicing factor 3B subunit 4	mRNA Processing	e	*A. thaliana*	Kundu et al., 2013
91	AtRACK1A	AT4G34620	SSR16, Small subunit ribosomal protein 16	Translation/Ribosome biogenesis	e	*A. thaliana*	Kundu et al., 2013
92	AtRACK1A	AT4G39200	S25-4, 40S Ribosomal protein	Translation	e	*A. thaliana*	Kundu et al., 2013
93	AtRACK1A	AT5G02960	S23-2, 40S Ribosomal protein	Translation	e	*A. thaliana*	Kundu et al., 2013
94	AtRACK1A	AT5G06360	S8e, Ribosomal protein family protein	Translation/Ribosome biogenesis	e	*A. thaliana*	Kundu et al., 2013
95	AtRACK1A	AT5G23740	RPS11-BETA, 40S Ribosomal protein S11-3	Translation	e	*A. thaliana*	Kundu et al., 2013
96	AtRACK1B	AT5G43960	NTF2, Nuclear transport factor 2 family protein with RNA Binding (RRM-RBD-RNP motifs) domain	Nucleocytoplasmic transport	d	*A. thaliana*	Dreze et al., 2011
97	AtRACK1B	AT5G48650	NTF2, Nuclear transport factor 2 family protein with RNA Binding (RRM-RBD-RNP motifs) domain	Nucleocytoplasmic transport	d	*A. thaliana*	Dreze et al., 2011
98	AtRACK1C	AT5G51190	ERF105, Ethylene-responsive transcription factor	Transcription	c	*A. thaliana*	Klopffleisch et al., 2011
**GROWTH, DEVELOPMENT, AND HORMONAL RESPONSE**							
99	AtRACK1A	AT1G08590	CLV1-Like leucine rich repeat transmembrane receptor-like protein kinase	Vascular development	e	*A. thaliana*	Kundu et al., 2013
100	AtRACK1A	AT1G78370	GSTU20, TAU 20 Glutathione S-transferase	Regulation of growth	e	*A. thaliana*	Kundu et al., 2013
101	AtRACK1A	AT2G02850	ARPN Plantacyanin	Development	e, f	*A. thaliana*	Kundu et al., 2013
102	AtRACK1A	AT2G14890	AGP9, Arabinogalactan protein 9	Meristem growth	e	*A. thaliana*	Kundu et al., 2013
103	AtRACK1A	AT3G07900	O-fucosyltransferase-like protein	Root hair cell differentiation	e	*A. thaliana*	Kundu et al., 2013
104	AtRACK1C	AT3G20830	AGC, cAMP-dependent, cGMP-dependent and protein kinase C family protein	Response to brassinosteroid and auxin	c	*A. thaliana*	Klopffleisch et al., 2011
105	AtRACK1B/AtRACK1C	AT4G35470	PIRL4, Plant intracellular ras group-related LRR 4	Gibberellin signaling	d	*A. thaliana*	Dreze et al., 2011
106	AtRACK1C	AT5G06110	DNAJ Domain; Myb-like DNA-binding domain	Cell division/Protein folding	c	*A. thaliana*	Klopffleisch et al., 2011
**OTHER AND UNKNOWN FUNCTIONS**							
107	AtRACK1A	AT1G18210	CML27, Putative calcium-binding protein	Unknown	e	*A. thaliana*	Kundu et al., 2013
108	AtRACK1C	AT1G22920	CSN5A, COP9 Signalosome complex subunit 5a, JAB1, AJH1	Photomorphogenesis	c	*A. thaliana*	Klopffleisch et al., 2011
109	AtRACK1A	AT1G23100	GroES-like protein	Protein folding	e	*A. thaliana*	Kundu et al., 2013
120	AtRACK1A	AT1G64230	UBC28, Ubiquitin-conjugating enzyme E2 28	Protein catabolic process	e	*A. thaliana*	Kundu et al., 2013
111	AtRACK1C	AT1G68410	PP2Cc, Protein phosphatase 2C 15 family protein	Unknown	c	*A. thaliana*	Klopffleisch et al., 2011
112	AtRACK1A	AT2G04900	Uncharacterized protein	Unknown	e	*A. thaliana*	Kundu et al., 2013
113	AtRACK1A	AT2G10940	Bifunctional inhibitor/lipid-transfer protein/seed storage 2S albumin superfamily protein	Lipid transport	e	*A. thaliana*	Kundu et al., 2013
114	AtRACK1A	AT2G18510	RRM1_SF3B4; RNA recognition motif 1 in splicing factor 3B subunit 4 (SF3B4)	Embryo dormancy	e	*A. thaliana*	Kundu et al., 2013
115	AtRACK1A	AT2G21045	Rhodanese-like domain-containing protein	Ion transport	e	*A. thaliana*	Kundu et al., 2013
116	AtRACK1C	AT2G22880	VQ Motif-containing protein	Response to UVB	c	*A. thaliana*	Klopffleisch et al., 2011
117	AtRACK1B/AtRACK1C	AT2G29080	FTSH3 protease/AAA+ ATPase	Protein catabolic process	c	*A. thaliana*	Klopffleisch et al., 2011
118	AtRACK1A	AT2G30105	LRR-UBQ, Leucine-rich repeats and ubiquitin-like domain-containing protein	Unknown	e	*A. thaliana*	Kundu et al., 2013
119	AtRACK1C	AT2G44310	Calcium-binding EF hand containing protein	Unknown	c	*A. thaliana*	Klopffleisch et al., 2011
120	AtRACK1A	AT2G44500	O-fucosyltransferase family protein	Unknown	e	*A. thaliana*	Kundu et al., 2013
121	AtRACK1A	AT2G46000	Uncharacterized protein	Unknown	e	*A. thaliana*	Kundu et al., 2013
122	AtRACK1C	AT2G47090	Zinc ion binding/nucleic acid binding	Unknown	c	*A. thaliana*	Klopffleisch et al., 2011
123	AtRACK1A	AT2G47590	PHR2, Photolyase/blue-light receptor 2	DNA repair	e	*A. thaliana*	Kundu et al., 2013
124	AtRACK1A	AT3G07565	Uncharacterized protein	Unknown	e	*A. thaliana*	Kundu et al., 2013
125	AtRACK1A	AT3G08690	UBC11, Ubiquitin-conjugating enzyme E2 11	Protein catabolic process	e	*A. thaliana*	Kundu et al., 2013
126	AtRACK1A	AT3G13520	AGP12, Arabinogalactan protein 12	Modified amino acid biosynthesis	e	*A. thaliana*	Kundu et al., 2013
127	AtRACK1A/AtRACK1B	AT3G26090	RGS1, Regulator of G-protein signaling 1	Sugar response	c, d, g	*A. thaliana*	Klopffleisch et al., 2011
128	AtRACK1C	AT3G56410	Uncharacterized protein	Unknown	c	*A. thaliana*	Klopffleisch et al., 2011
129	AtRACK1A	AT4G27960	UBC9, Ubiquitin conjugating enzyme	Protein catabolic process	e	*A. thaliana*	Kundu et al., 2013
130	AtRACK1A	AT4G28030	GCN5-Related N-acetyltransferase (GNAT) family protein	Metabolism	e	*A. thaliana*	Kundu et al., 2013
131	AtRACK1C	AT4G37540	LBD39, LOB domain-containing protein 39	Membrane fluidity	c	*A. thaliana*	Klopffleisch et al., 2011
132	AtRACK1B/AtRACK1C	AT5G03240	UBQ3, Polyubiquitin 3	Protein catabolic process	a	*A. thaliana*	Kim et al., 2013
133	AtRACK1A	AT5G04750	Putative F1F0-ATPase inhibitor protein	Unknown	e	*A. thaliana*	Kundu et al., 2013
134	AtRACK1A	AT5G11500	Uncharacterized protein (DUF814)	Unknown	e	*A. thaliana*	Kundu et al., 2013
135	AtRACK1A	AT5G48180	NSP5, Nitrile specifier protein 5	Catabolic processes	e	*A. thaliana*	Kundu et al., 2013
136	AtRACK1A	AT5G52430	Hydroxyproline-rich glycoprotein family protein	Unknown	e	*A. thaliana*	Kundu et al., 2013
137	AtRACK1A	AT5G53300	UBC10, Ubiquitin-conjugating enzyme E2 10	Protein catabolic process	e	*A. thaliana*	Kundu et al., 2013
138	AtRACK1C	AT5G65780	ATBCAT-5, Branched-chain-amino-acid aminotransferase 5	Metabolism	c	*A. thaliana*	Klopffleisch et al., 2011

*In order to obtain homogeneous data, the ligand names and related process were assigned based on the gene information from each ID at the NCBI database. a, Affinity Capture; b, Co-Immunoprecipitation; c, cDNA Library screen by YTH assay; d, One by one YTH assay; e, cDNA Library screen by suYTH system; f, One by one suYTH assay; g, in planta or protoplasts BiFC; and h, Split firefly luciferase complementation (SFLC)*.

## Hormonal signaling: species- and tissue-specific responses

Since the discovery of *arcA*, the first plant RACK1 homolog sequence from a subtraction library of BY2 cultured cells subjected to auxin treatment (Ishida et al., 1993), it was reported that its expression was regulated (induced) by the hormone. Interestingly, this expression was exclusively stimulated by auxins but not by cytokinins, abscisic acid (ABA), ethylene or heat (Ishida et al., 1996). It is interesting to note that RACK1 has been found in stoichiometric quantities on the small subunit of crystallized eukaryotic ribosomes (Gibson, 2012), and that it has also been implicated in protein translation and ribosome binding in plants (see below Chang et al., 2005; Ullah et al., 2008; Guo et al., 2011a,b). Thus, it is tempting to suggest that plant RACK1 may be able to regulate its own expression via hormonal regulation. After Ishida et al. (1993) reported auxin-regulated expression of *arcA*, several other reports implicated RACK1 in hormonal signaling pathways. For example, Nakashima et al. (2008) reported that auxin, ABA and methyl jasmonate, induced rice *RACK1A* expression. As in tobacco cultured cells, in rice cultured cells the auxin was also an inducer of Os*RACK1*; however, unlike the case of *ArcA*, ABA did stimulate *OsRACK1A* expression (Ishida et al., 1996; Nakashima et al., 2008). In a separate similar report on *Zea mays*, growing seedlings were subjected to ABA treatment while methyl jasmonate was sprayed to the expanding leaves of growing plants. In both cases, *ZmRACK1* expresssion was induced and only a slight difference between both hormonal treatments was observed (Wang et al., 2014). These results further suggested that RACK1 indeed participated in hormonal signaling, albeit, under different regulation from species to species.

Early reports included the implication of Msgbl, the RACK1 homolog from *M*. *sativa*, in hormone-mediated cell division since the transcript was preferentially located to dividing cells of nodule primordia and meristem (McKhann et al., 1997). However, contrary to *arcA*, which was exclusively induced by auxin (Ishida et al., 1996), Mgsbl was induced by cytokinin treatment of roots (McKhann et al., 1997). This work suggested that plant RACK1 was involved in several hormone-mediated pathways. Most recent work has been carried out on *A*. *thaliana RACK1* isogenes *AtRACK1A, B*, and *C*, although a functional redundancy derived from the high sequence conservation was expected and subsequently confirmed (Guo and Chen, 2008). The involvement of RACK1 in multiple plant hormonal pathways was confirmed by the study of hormonal treatments and developmental processes of loss-of-function *Arabidopsis rack1a* mutants (Chen et al., 2006; Fennell et al., 2012). The *rack1a* mutants displayed defects in seed germination, flowering, and production of leaves. The mutants also displayed a number of altered responses to hormones such as: (a) reduced sensitivity to gibberellin and brassinosteroid during seed germination; (b) hypersensitivity to ABA during seed germination and early seedling development; and (c) hyposensitivity to auxin in adventitious and lateral root formation (Chen et al., 2006). The authors concluded that AtRACK1A participated in multiple signal transduction pathways during plant developmental processes. These data were further substantiated by the report that *Arabidopsis rack1a single, and rack1a/rack1b or rack1a/rack1c* double mutants, were hypersensitive to ABA in developmental processes that included seed germination, cotyledon greening and root growth (Guo et al., 2009a). In addition, the mutants lost water more slowly from the rosettes than the wild type, and were hypersensitive to high concentrations of NaCl during seed germination. On the other hand, plants overexpressing AtRACK1A displayed ABA insensitivity. The authors concluded that *Arabidopsis* RACK1 was a negative regulator of ABA responses and that the three *AtRACK1* genes act redundantly to regulate such ABA responses. Interestingly, the expression of all three *AtRACK1* genes was down-regulated by ABA, an effect contrary to the stimulation of expression by auxin (Ishida et al., 1993) or cytokinin (McKhann et al., 1997). In a later study, it was found that all three AtRACK1 proteins interacted physically with the two eIF6 protein isoforms in *Arabidopsis* (Guo et al., 2011a), and that ABA down-regulated the expression of both *AtRACK1* and *elF6* transcripts. These data further suggested that plant RACK1 may be able to regulate its own expression in a hormonally-regulated feedback loop.

Interestingly, it was found in a different work that the AtRACK1A isoform seems to specifically mediate gibberellin signaling, at least in a D-allose mediated inhibition pathway (Fennell et al., 2012). When gibberellin was applied to *Arabidopsis* seeds, a significant up-regulation of AtRACK1A:GFP expression in the embryo root tip region of seedlings was observed after 72 h. The opposite effect was observed by treatment with the rare sugar D-allose, which is an inhibitor of seed germination (Fennell et al., 2012). Further analysis on *Arabidopsis rack1a* knockout mutants showed a significantly higher hypersensitivity to D-allose inhibition of germination compared to wild type seeds, and this inhibition was not counteracted by the addition of gibberellin. Finally, in seeds harboring a functional RACK1A in a *rack1b*/*rack1c* double mutant background, neither D-allose nor D-allose plus gibberellin significantly affected seed germination. These data suggested that D-allose negatively regulated seed germination and gibberellin-mediated early seedling development through the inhibition of RACK1A expression (Fennell et al., 2012). These results also indicated that specific plant RACK1 isoforms may be involved in hormonal responses regulating developmental processes in different fashion.

Hormonal relationships to RACK1 in symbiotic legumes have been also carried out (McKhann et al., 1997; Islas-Flores et al., 2009, 2011, 2012). In the smaller *P*. *vulgaris RACK1* (*PvRACK1*) gene family, treatment of bean seeds with the synthetic auxins 2,4-D (2, 4-Dichlorophenoxyacetic acid) and IAA (Indole-3-acetic acid) showed that auxin negatively controlled *PvRACK1* transcript accumulation during germination. It was found that the maximum transcript accumulation of *PvRACK1* was at 32 h of germination without treatment. However, this accumulation was delayed 8 h from the control with synthetic auxin treatment (Islas-Flores et al., 2009). These findings suggested a weak negative regulation of *PvRACK1* transcription mediated by auxin. This conclusion was supported by the fact that the auxin transport blocker Naphthylphthalamic Acid (NPA) did not have any effect on the level of *PvRACK1* transcript, which was consistent with a putative blocking of the auxin transport to target sites (Islas-Flores et al., 2009). In a subsequent work, it was demonstrated that the *PvRACK1* transcript accumulation was induced by ABA, cytokinin, and gibberellin during root development. However, contrary to the above effect seen on *P. vulgaris* germination, the transcript was also induced by auxin on developing roots. It was shown that the transcript induction was higher with auxin, gibberellin and ABA than with cytokinin (Islas-Flores et al., 2011, 2012). Cytokinin induction of *RACK1* expression in roots of other legume was also documented in the previous report by McKhann et al. (1997), in *M. sativa*. Thus, hormonal signaling mediated by RACK1 on developing legume roots suggested its involvement in symbiotic processes (see below). Some of the provided data on the hormonal signaling-RACK1 relationship has also been discussed in a previous review (Zhang et al., 2013). Taken together, the results reported so far provide compelling evidence that plant RACK1 participates in hormonal signaling through regulation of protein expression. Its own expression, in a likely array of feedback loops, would also be regulated by phytohomones in tightly regulated signaling networks critical for fundamental plant developmental processes.

## Cell proliferation and plant development

RACK1 has been related and even suggested as marker for mammalian cell proliferative processes since early reports of its overexpression in breast, lung and hepatocellular carcinoma (reviewed in Li and Xie, 2015). Following this association, Islas-Flores et al. (2011) inferred that plant RACK1 could be involved in cell proliferation processes in symbiotic root nodules. Using the model of *P*. *vulgaris* root nodules, and with previous evidence that suggested that its genome only harbored one *PvRACK1* gene (Islas-Flores et al., 2009), they used an RNAi approach to silence its expression and follow the root nodule phenotype during development. It was observed that, as expected, silencing of *PvRACK1* expression led to a reduced number and size of the nodules compared to controls (Islas-Flores et al., 2011). They also observed a reduced red-brown coloration of *PvRACK1*-knockdown nodules with respect to controls. A closer examination of the cell ultrastructure revealed that *PvRACK1* knockdown prevented a proper formation of the symbiosome and impaired cell expansion, which resulted in a reduced cell size of both infected and non-infected cells (Islas-Flores et al., 2011). These data were also consistent with earlier findings where the *RACK1* transcript was preferentially located to dividing cells of nodule primordia and meristem in *M*. *sativa* (McKhann et al., 1997). These results suggested that plant RACK1 is also involved in cell proliferation and expansion directly related to symbiotic processes.

RACK1-induced developmental defects were studied in *A*. *thaliana* with T-DNA insertion mutants. The *rack1a* loss-of-function mutation in *Arabidopsis* led to the impairment of multiple developmental processes that included seed germination, leaf production, and flowering (Chen et al., 2006). It was later reported (Guo and Chen, 2008), that loss-of-function mutations in either *rack1b* or *rack1c* did not confer the same developmental defects and phenotypes were apparently normal, indicating that *AtRACK1A* could functionally complement those mutations. Interestingly, either *rack1b* or *rack1c* mutation enhanced the developmental defects caused by the *rack1a* mutation in double mutant analyses. Furthermore, severe effects on development and lethality were observed in the *rack1a*/*rack1b*/*rack1c* triple mutants. The authors concluded that *RACK1* genes function in an unequally redundant manner, critically and tightly regulating plant development (Guo and Chen, 2008). More recently, Zhang et al. (2014) reported an interesting work linking OsRACK1 to rice seed germination. They first observed that in wild type rice seeds, the *OsRACK1A* gene was highly expressed while *OsRACK1B* was poorly expressed. They assessed its role in the control of seed germination and observed that OsRACK1A underexpressing seeds showed a significant delay in germination and a decrease in their germination rate compared to the wild type. These knockdown mutants also displayed an increased sensitivity to an ABA-induced germination delay compared to wild type or OsRACK1A overexpressing seeds (Zhang et al., 2014). These results indicated that OsRACK1A positively regulated seed germination in rice, similar to the first reports on the effect of RACK1 on *Arabidopsis* germination (Chen et al., 2006). Taken together, these reports clearly highlight a critical role of RACK1 in cell proliferation and developmental processes in plants. Zhang et al. (2014) proposed a mechanism in which OsRACK1A positively regulates seed germination through enhancing ABA catabolism and stimulation of H_2_O_2_ production, and that they both interact to regulate seed germination. More recently, a direct interaction between AtRACK1 and Gβ in *Arabidopsis* upon activation of a pathogen defense signaling pathway was reported (Cheng et al., 2015). Although, some direct RACK1 interactors have been found and these have revealed some clues regarding the impact they may have on developmental processes, many more are predicted yet to be discovered. These RACK1 interactors will be discussed in detail below.

## Innate immunity and ROS production

RACK1 is involved in innate immunity responses in plants through the formation of immune complexes. This aspect of RACK1 function is discussed in detail in a parallel review within this topic and we will only mention the most relevant and recent findings. Innate immunity in rice is regulated by a complex of regulatory proteins located at the plasma membrane. This assembly is composed of OsRACK1, Rac1, RAR1, SGT1, Rboh and the two HSP proteins, HSP90 and HSP70 (Thao et al., 2007; Nakashima et al., 2008). It was found that RACK1 participates in this complex by binding to Rac1, RAR1, SGT1, and Rboh but not HSP90 (Nakashima et al., 2008). Rac1 transcriptionally and post-transcriptionally regulates RACK1 and vice versa in a feedback loop. A model has been proposed in which, when a rice plant is attacked by pathogens (i.e., rice blast fungus), Rac1 and/or RACK1 is/are activated and the immune complex is formed. This newly formed immune complex can then directly regulate the immune response through HSP70 and HSP90, or interact with the N-terminus of RbohB to trigger the production of ROS and fight the pathogen (Nakashima et al., 2008; Kawano et al., 2014). More recently, Cheng et al. (2015) reported that in *Arabidopsis*, all three RACK1 subunits also serve as scaffolds for the MAPK pathway through binding to the Gβ subunit upon activation of the MAPK cascade by a pathogen-secreted protease. This cascade resulted in the activation of the immune response although further downstream defense responses were not investigated. It is possible that in this instance, ROS are elicited to fight the invader, as it has been proposed to occur in rice (Nakashima et al., 2008). The authors concluded that a mechanistically distinct immune signaling must occur in plants compared to yeast or mammals. In the latter, Ste5 or β-arrestin scaffold MAPK's, after upstream GPCRs activation, whereas plants do not possess the corresponding orthologs (Cheng et al., 2015). Recently, RACK1 expression was linked to pathogen responses that led to ROS production in Maize (Wang et al., 2014). These authors observed an increased level of *ZmRACK1* expression upon ABA or methyl jasmonate treatment and hypothesized that in maize, a similar innate immunity response to that occurring in rice (Nakashima et al., 2008), could exist. In agreement, *ZmRACK1* overexpression resulted in a 2.5–3-fold increase in expression levels of the pathogenesis-related protein genes *PR-1* and *PR-5*, and the protein was shown to interact with RAC1, RAR1 and SGT1. These proteins were present in the Rac1 (a ROP/RAC small GTPase) immune complex as determined by the Yeast Two Hybrid (YTH) assay (Wang et al., 2014). It was also observed that ROS production was higher in seedlings and leaves from overexpressing lines than in the wild-type. These results were in direct analogy with the immune response in rice where an equivalent complex is formed and ROS production ensues (Kawano et al., 2014).

G-protein signaling has been linked to ROS generation in *Arabidopsis* guard cells as *gpa1* mutants were impaired in the production of ROS in response to ABA (Zhang et al., 2011). In addition, Gudesblat et al. (2007) reported that MPK3 kinase acts downstream of ROS production in guard cell ABA signaling. Thus, pathogen-activated defense responses in plants may occur through similar mechanisms to those utilized for hormone-mediated ROS production. This is consistent with the model proposed by Kawano et al. (2014), where G-protein functions upstream of OsRac1 in the early steps of rice defense signaling.

## RACK1 also appears on the scene of stress responses

RACK1 has been implicated in a variety of responses elicited by both biotic and abiotic stress in yeast (Núñez et al., 2009), worms (Ziegler et al., 2009), mammalian cells (Arimoto et al., 2008), and plants. Since ABA plays a major role in drought and saline stress, and Guo et al. (2009a) had shown that *rack1a* single, *rack1a*/*rack1b, rack1a*/*rack1c*, and *rack1b*/*rack1c* double loss-of-function mutants exhibited hypersensitivity to ABA, they tested seed germination on the same mutant lines subjected to salt stress. The results showed a direct relationship between ABA sensitivity and salt stress sensitivity in seed germination. In their assays, *rack1a* single and *rack1a*/*rack1b* or *rack1a*/*rack1c* double mutants, were severely affected during seed germination under salt stress. On the other hand, the *RACK1A* overexpressing plants showed a hyposensitivity to the salt treatment in the germination assay. These results directly linked AtRACK1 to stress responses in *Arabidopsis* (Guo et al., 2009a). Unfortunately, no determination of the effect of salt or other types of stress on the expression of the *AtRACK1* genes was carried out on germinating *Arabidopsis* wild type seeds to directly test the stress effects on *AtRACK1* regulation.

In a separate work, using *P*. *vulgaris* plant roots transformed with *A*. *rhizogenes* carrying a *PvRACK1* overexpression construct, severe damage and necrosis was observed when these were subjected to heat stress. Heat-shocked transformed seedlings showed systemic necrosis at 4–5 days post-inoculation, no callus formation at the inoculation zone, and interrupted progression to transgenic root formation (Islas-Flores, 2011; Islas-Flores et al., 2012). These observations suggested that the overexpression of the *PvRACK1* gene in *P*. *vulgaris*, caused a severe imbalance in the RACK1-mediated signaling cross-talk leading to an enhanced effect of heat stress, which resulted in lethality. The exact mechanisms and RACK1 interactors underpinning this enhanced stress effect remain to be studied.

Subsequently, Kundu et al. (2013) identified oxidative, drought and saline stress RACK1 ligands when AtRACK1A was used as a bait to screen an *A. thaliana* inflorescence cDNA library by the split-ubiquitin Yeast Two-Hybrid system (suYTH). Identified ligands corresponded to CSD1 (Superoxide dismutase [cu-Zn]), MYC2 (Transcription factor), GSTU19 (TAU 19 Glutathione S-transferase), Fructose-bisphosphate aldolase 3, ATPQ (ATP synthase subunit D), RAN-1 (GTP-binding nuclear protein Ran-1; RAS-related nuclear protein-1), WNK8 (WITH NO LYSINE (K) KINASE 8; Serine/threonine-protein kinase), and LTP3 and LTP4 (Non-specific lipid-transfer proteins 3 and 4; Kundu et al., 2013; Table 1). These results also linked RACK1 function to diverse types of stress including hypoxia, cold, and starvation.

More recently, Olejnik et al. (2011) found that AtRACK1A binds to AtNUDT7. AtNUDT7 is a pyrophosphatase that hydrolyzes NADH and ADP-ribose *in vitro*, and plays a role in the response to biotic and abiotic stress. The authors proposed that the AtRACK1A-AtNUDT7 interaction negatively regulates the cellular level of ROS and the cellular defense pathway. This work uncovered new players in a novel pathway in which RACK1 scaffolded interactors that are involved in stress responses in *Arabidopsis*. Similarly, RACK1 expression was linked to biotic stress imposed by pathogen responses in rice and maize (Nakashima et al., 2008; Wang et al., 2014). Rice and maize plants overexpressing OsRACK1A or ZmRACK1, respectively led to a reduction in leaf symptoms caused by the fungi *Magnaporthe grisea* and *Exserohilum turcicum* (Pass.), respectively. The extension of the leaf chlorosis and necrosis was statistically and significantly lower in overexpressing OsRACK1A or ZmRACK1 leaves than in the wild type. Unfortunately, no assessment of the stress effect imposed by the pathogens on *OsRACK1A* or *ZmRACK1* expression was carried out, although the *ZmRACK1* transcript was up-regulated in leaves pre-treated with ABA and methyl jasmonate (Wang et al., 2014). The above results implicate both, plant RACK1 in the activation of biotic and abiotic stress responses, and ABA in the regulation of these responses. The exact mechanisms of action and clarification of the opposing effects in different plant species await further study.

## Does RACK1 regulate the microRNA pathway?

MicroRNAs (miRNAs) play significant roles in living systems by modifying most protein coding transcripts at the post-transcriptional level. miRNAs are a class of 21 nucleotide non-coding small RNAs, simultaneously targeting many transcripts and fine-tuning the expression of genes (Carrington and Ambros, 2003; Bartel, 2004; Nunez et al., 2013). Although the process of miRNA-mediated regulation of gene expression shows similarities in plant and animal systems, there are clear distinctions in terms of the identity of the regulatory proteins and the cellular sites of miRNA biogenesis. In animals, these tiny (~21 nucleotide) miRNAs are involved in developmental and pathological processes. In plants, miRNAs participate in growth, flowering and development by regulating hormone signaling, nutrient sensing, stress responses and immunity against pathogen invasion (Ding et al., 2013; Jin et al., 2013). While plant and non-plant miRNAs are both transcribed by the RNA polymerase II, their biogenesis differs in terms of location. Metazoan miRNAs are processed at two locations, the cytoplasm and the nucleus. In the nucleus, pri-miRNAs are processed by DROSHA and DIGEORGE SYNDROME CRITICAL REGION 8 (DGCR8), and the processed pre-miRNAs are then further processed by DICER in the cytoplasm (Lee et al., 2003; Kim, 2005). Unlike their animal counterpart, plants do not have DROSHA and DGCR8 to process the pri-miRNA to the pre-miRNA. Instead, another RNase III-like protein known as DICER-LIKE 1 (DCL1), in conjunction with the SERRATE (SE) or HYPONASTIC LEAVES 1 (HYL1), process the pri-miRNAs into the mature miRNAs, which occur exclusively in the nucleus. The mature miRNAs are loaded onto the ARGONAUTE (AGO) effector complexes-miRNA-induced complex (miRISC), which regulates the target mRNAs for degradation or repression of translation (Kurihara and Watanabe, 2004).

To date, three different reports have implicated RACK1 in the miRNA pathway in *C*. *elegans*, humans, and *Arabidopsis* (Jannot et al., 2011; Speth et al., 2013; Speth and Laubinger, 2014). In plants it was found, through a YTH assay, that RACK1 interacts with the SE protein to regulate the mature miRNA biogenesis in the nucleus (Speth et al., 2013; Speth and Laubinger, 2014). Subsequently, the interaction was confirmed *in vivo* by the Bimolecular Fluorescence Complementation (BiFC) assay. It was found that such interaction took place in distinct sub-nuclear foci D-bodies, where SE was previously reported to localize (Fang and Spector, 2007). Further support came with the report that miRNA accumulation in *rack1* loss-of-function mutants was globally decreased causing de-repression of the target mRNAs (Speth et al., 2013; Speth and Laubinger, 2014). It was also reported that some pri-miRNAs accumulated at higher levels in *rack1* mutants, suggesting that RACK1 affects the processing and transcription/stability of certain pri-miRNAs in plants (Speth et al., 2013; Speth and Laubinger, 2014). Moreover, aberrant non-canonical miRNAs were more abundant in *rack1* mutants, indicating that RACK1 is also involved in ensuring precise processing (Speth et al., 2013; Speth and Laubinger, 2014). Plant pri-miRNAs and pre-miRNAs are cleaved in the nucleus by the same RNase III enzyme DICER-LIKE 1 (DCL1). Therefore, *Arabidopsis* RACK1 may be required for steps upstream of, or with DCL1, to affect pri-miRNA processing or stability.

Although RACK1 interaction with SE indicates a role in miRNA biogenesis, it is not difficult to envision a RACK1 role independent of miRNA biogenesis. For example, Otsuka et al. (2011) reported, in hepatocellular carcinoma, that a few miRNAs displayed impaired silencing upon RACK1 depletion with no detectable changes in their overall levels (Otsuka et al., 2011). However, lower amounts of these miRNAs were detected in Ago2-containing complexes, leading the authors to propose that RACK1 functions after miRNA maturation and is required to load mature miRNAs into miRISCs. Similarly, Jannot et al. (2011) observed that in *C. elegans* and human cells depletion of RACK1 impaired miRNA regulation and reduced the amount of AGO associated with the polysome (Jannot et al., 2011). They proposed that RACK1, as an AGO-interacting protein, facilitates the recruitment of miRISC to the ribosome. Interestingly, Speth and Laubinger (2014) have shown that *Arabidopsis* AtRACK1 and AGO1 are part of a common complex outside the ribosome, and that they co-localize in the nucleus and the cytoplasm. As AtRACK1 has been shown to regulate pri-miRNA processing in the nucleus, it is likely that free, rather than ribosome-bound RACK1, is involved in the regulation of miRNA biogenesis (Speth et al., 2013; Speth and Laubinger, 2014). Differences in localization and binding partners may explain the distinct functions of RACK1 in miRNA biogenesis and miRISC function. Ribosome-bound RACK1 may help recruit miRISCs to the ribosome, while free RACK1, perhaps with associated AGO, may play roles in miRNA biogenesis in the nucleus and/or the cytoplasm (Chu et al., 2014).

Whether RACK1 binds miRNA directly is not resolved yet. Although RACK1 is predicted to bind the ribosome by primarily anchoring to the 18S ribosomal RNA, the mechanism of binding to the miRNA may be mediated by interaction with other anchoring protein(s) like SE or HYL1.

Given the evidence that RACK1 is a negative regulator of the stress hormone ABA signaling, it is not far-fetched to suggest that RACK1 regulates this pathway possibly through miRNA regulation. Indications that miRNAs participate in the ABA response were first provided by the isolation of ABA-hypersensitive mutants impaired in any of the several key genes of the miRNA biogenesis pathway, *HYL1, DCL1, HEN1, SE*, and *HASTY* (Ding et al., 2013). The *hyl1* mutant was shown to be hypersensitive to ABA during *Arabidopsis* germination (Lu and Fedoroff, 2000). Drought-induced miRNAs down-regulate their target mRNAs which may encode negative functional proteins involved in the drought response. Conversely, other miRNAs are down-regulated, leading to the accumulation of their target mRNAs that contribute positively to stress adaptation. The precise identification of the miRNAs and their targets in the *rack1* mutants would definitely allow to pinpoint the exact role of RACK1 in the miRNA-mediated drought responses.

## Are the ribosome-bound RACK1 ligands lost in translation?

**Figure 2:**
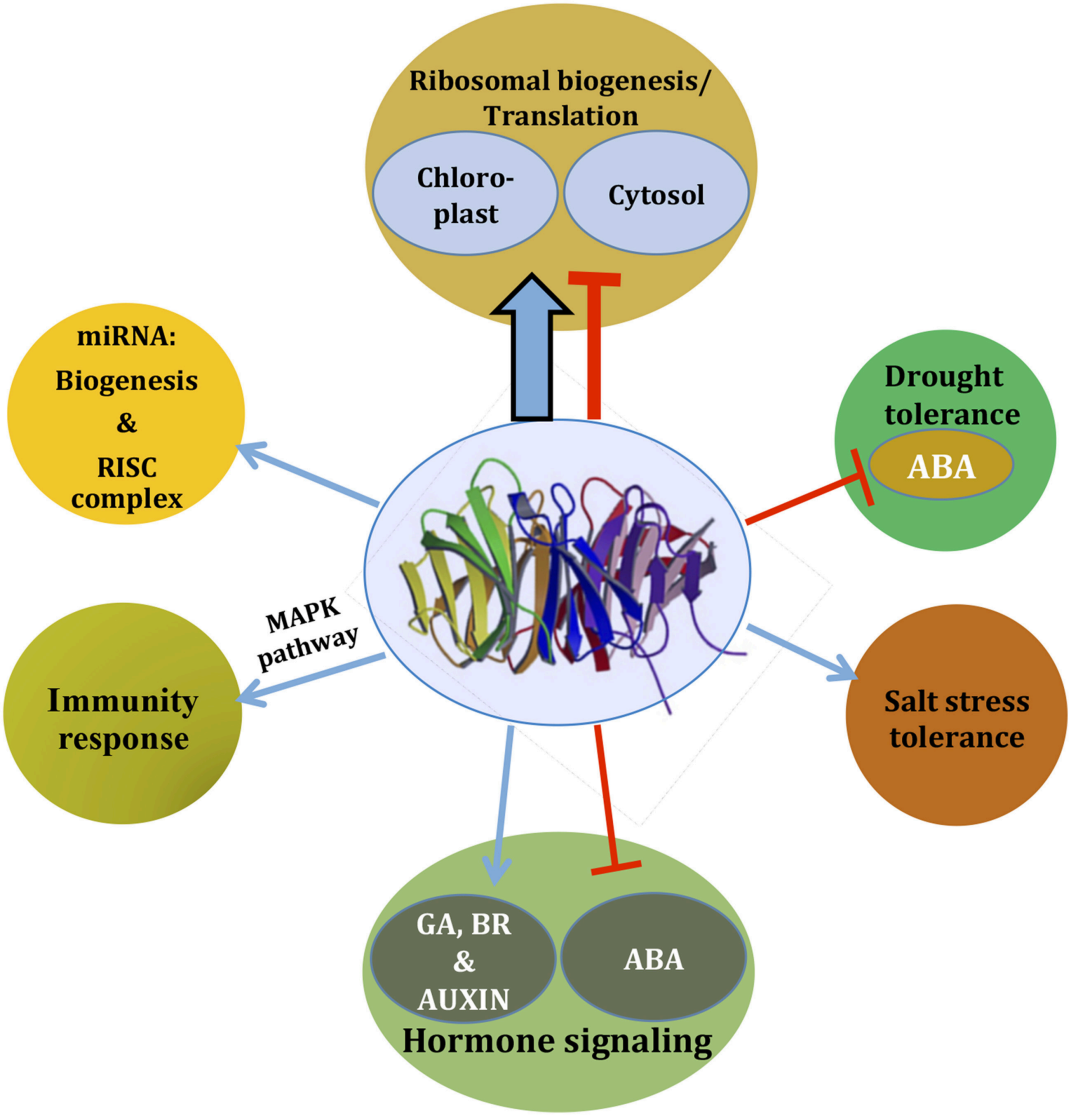
**As an integrator of signaling, RACK1 regulates diverse cellular signaling pathways**. Based on the available literature, a comprehensive view is presented. Depending on the pathways, RACK1 acts either as a positive regulator (blue arrow) or as a negative regulator (red inhibitory arrow), most likely through mediating protein-protein interaction. The thicker arrow denotes a predominant role of RACK1 in protein translation and ribosome biogenesis. Note that the pathways may not always act in a unidirectional way as they often cross-talk. See text for further detail.

Although RACK1 has been implicated in many stress signaling pathways, the *in silico* study of RACK1 inside the GENEVESTIGATOR database indicates that RACK1 predominantly regulates protein translation and ribosome biogenesis (Guo et al., 2011b). Through a careful analysis of the online data and co-expression studies, Guo et al. (2011b) found that the protein synthesis and ribosome biogenesis function of RACK1 is regulated by the stress hormone ABA. Loss in the relative abundance of 60S ribosome subunits and 80S ribosome in the *rack1a*/*rack1b* double mutants indicated a role of RACK1 in the ribosome biogenesis. Such predominant role of RACK1 in protein translation and ribosome biogenesis is, therefore, represented by a thicker arrow in Figure 2. In addition, by virtue of the RACK1 predominant localization on the ribosome in different species, a role for the protein in the regulation of global translation through ribosome biogenesis and interaction with regulatory proteins has been envisioned (Shor et al., 2003; Gerbasi et al., 2004; Nilsson et al., 2004; Sengupta et al., 2004; Chang et al., 2005; Giavalisco et al., 2005; Yu et al., 2005; Regmi et al., 2008; Coyle et al., 2009; Armache et al., 2010; Guo et al., 2011a,b). In a recent report, it was also revealed that *Arabidopsis* RACK1A, in conjunction with other ribosomal proteins, controls the upstream Open Reading Frame (uORF)-mediated protein translation of the key transcription factor SAC51 to regulate growth and development (Kakehi et al., 2015). Using Cryo-EM, Sengupta et al. (2004) found that fungus RACK1 was located on the head region of the 40S subunit, in the immediate vicinity of the mRNA exit channel. Later, Coyle et al. (2009) provided evidence that *S. cerevisiae* RACK1, Asc1 functions on the ribosome, implying a physical link between the eukaryotic ribosome and cell signaling pathways *in vivo*. Chang et al. (2005), in a proteomic analysis of ribosomal proteins, first reported the identification of RACK1 protein from the *Arabidopsis* ribosome, where it was associated with both the 40S ribosome subunit and polysomes. It has been suggested that, although ribosome-bound RACK1 might directly regulate translation *per se*, its function as a ribosomal protein was likely linked to its capacity to recruit a particular cohort of RACK1-associated proteins such as activated PKCβII in animals (Adams et al., 2011). However, the lack of *bona fide* PKC isoforms from the plant kingdom indicate that other kinases may play a similar role in regulating RACK1-mediated ribosome translation processes. On the other hand, a suggested model for the role of RACK1 protein in ribosome assembly in non-plants entails phosphorylation of eIF6 by PKC. The latter as a result of interaction with eIF6 and PKC bound to a nearby 40S subunit via RACK1 (Sengupta et al., 2004). The report that *Arabidopsis* RACK1 directly interacts with eIF6 implicates plant RACK1 in mediating the ribosome assembly in a similar way as observed with their counterparts in mammalian cells (Guo et al., 2011a). The superposition of the deduced crystal structure of *Arabidopsis* RACK1A onto the yeast RACK1-40S ribosome model (PDB ID code 1ARI) supports the notion that the top rim of RACK1A is in contact with the ribosome allowing the bottom rim to interact with regulatory proteins (Ullah et al., 2008). The lack of any structural data on plant ribosome-bound RACK1 makes difficult to support the claim that both, the mammalian and plant RACK1 proteins function in the same mechanistic way during the regulation of protein translation. It appears even more difficult, to extrapolate similar pathways of RACK1-ribosomal complexes that are modulated under stress in mammalian cells (Arimoto et al., 2008). When these cells enter stress by hypoxia or heat shock (type 1 stress), the formation of stress granules is induced. Stress granules are molecular aggregates of stalled translation pre-initiation complexes (that prevent the accumulation of mis-folded proteins), which also sequester RACK1. This RACK1 immobilization, in turn, suppresses the activation of the MTK1-SAPK pathway leading to apoptosis, which would otherwise be induced under conditions of type 2 stress (X-ray or genotoxic drug exposure; Arimoto et al., 2008). Plants do not display, at least phenotypically, any similar response under the various stress conditions. This is most likely due to the plasticity and ancestry of plant genomes brought about by the evolution pressure that the incapability of movement has imposed. The mechanisms of regulation of translation in plants under stress are more likely to be under hormonal control, in which RACK1 also actively participates. For example, since RACK1 is known to be a negative regulator of the stress hormone ABA, it is possible that plants are also able to modulate global protein translation through RACK1-ribosomal associations under stress conditions. The putative critical kinases and phosphatases key for regulation through phosphorylation/dephosphorylation events, whether or not present in multimolecular complexes with RACK1, remain unidentified.

Finally, another feature that sets apart plant RACK1 proteins from their non-plant counterparts, is their putative role in the regulation of the chloroplastic ribosome-based translation process. Kundu et al. (2013) reported that RACK1A, the predominant isoform in *Arabidopsis*, physically interacts with dozens of cytosolic and chloroplastic ribosomal proteins. Important among them is the RPL12-A protein which contains a signal sequence that exclusively localizes the protein to the chloroplast. In addition, RACK1A is also found to interact with a member of Ribosomal protein L36a/L7-3 family protein that is also reported to be localized in the chloroplast (Kundu et al., 2013). Interestingly, it was reported that the spinach chloro-ribosome L7-3/L12-A proteins in combination with L1, form the base stalks of the 70S chloro-ribosome as observed by Cryo-EM. These base stalks were larger than those of the *Escherichia coli* 70S ribosome, one of various features that distinguished them from one another (Sharma et al., 2007). In view of the RACK1 associations with putative chloroplast-localized ribosomal proteins, it is tempting to speculate that RACK1 may regulate the translation process by either affecting the chloro-ribosome assembly and/or stability, or by directly regulating the translation process.

RACK1A is also reported to interact with the major components of photosynthetic proteins translated by the chloro-ribosomes which include photosystem I and II components and the large subunit of RuBisCo-associated proteins. Since chloro-ribosomes control chloroplastic translation in a light dependent manner, it will be interesting to investigate whether plant RACK1 is able to regulate the photosynthetic protein abundance of the chloroplast. This would have a direct effect on regulating the photosynthesis-based total growth and development of a plant.

## RACK1-interacting ligands, some solid evidence, and some enigma

Structurally, RACK1 presents multiple conserved surface residues contained on the WD-40 repeats, where these binding sites allow multiple interactions and set the bases of its scaffolding characteristics. Although, it is capable to form homodimers, it is also found as a free monomer allowing to form hetero-complexes with an expanding number of partners. It is well known that RACK1 has a large number of direct ligands; for example, in metazoans over 90 ligands have been reported as RACK1 interactors (Adams et al., 2011). RACK1 binding partners have been identified in several intracellular fractions, where they are transported and placed in proximity to their modifying enzymes or substrates.

Plant RACK1 presents two conserved surface regions (region 1, blades 1–4; and region 2, blades 5 and 6) believed to mediate potential protein–protein interaction sites (Ullah et al., 2008). Probable interacting partners were proposed based on the comparison of residues contained in these regions, with known residues that mediate interaction with characterized ligands (Dell et al., 2002; Chen et al., 2004; Ullah et al., 2008). In addition, RACK1 has a structural and translational function as it has been documented that it forms part of the ribosome in metazoans and plants, and it is coexpressed with up to 80% of ribosome proteins (Guo et al., 2011b). RACK1 has been identified on the head of the 40S ribosomal subunit were its conserved region 1 directly interacts with the helices 39 and 40 of the 18S rRNA, and the ribosomal protein S2p, while the conserved region 2 is exposed for interaction and recruiting of binding partners such as PKC (Nilsson et al., 2004; Sengupta et al., 2004; Armache et al., 2010). Consequently, plant RACK1 should also be found in the ribosome for recruiting PKC to phosphorylate eIF6 as in animal cells (Ceci et al., 2003). However, it is well documented that plant genomes lack this type of kinase (Guo et al., 2011b) and so far no equivalent plant kinase to fulfill a parallel function has been found. Therefore, since direct comparison of RACK1 interacting ligands in animal cells cannot be extrapolated to plant cells, alternative strategies to unequivocally identify plant RACK1 interactors have been necessary. For example, approaches that demonstrate direct physical interaction such as the yeast two hybrid (YTH) and BiFC assays, confirmed interaction of AtRACK1A, B, and C with eIF6A and B (Guo et al., 2011b). These strategies confirmed, in part, what was observed in metazoans, as well as the functional involvement of plant RACK1 in protein translation and ribosome stability. It is important to highlight that, to date, still no plant kinase equivalent to animal PKC has been identified in those complexes so how the initiation of translation is regulated in plants remains an open question. The identification of *Oryza sativa* RACK1A (OsRACK1A) as part of the immune complex interacting with Rac1, Rboh, RAR1, and SGT1 (Nakashima et al., 2008) revealed the first plant RACK1 ligands, which had remained elusive for many years. This was achieved by means of a Rac1 affinity column in a search for Rac1 ligands in an assay using prior activation by a sphingolipid elicitor. OsRACK1A interaction was further confirmed by the YTH and suYTH assays. While OsRACK1B bound to a less extent to Rac1, the binding of the other Rac isoforms, Rac3, Rac5, Rac6, and Rac7 to OsRACK1A was also confirmed. Furthermore, the direct interaction of OsRACK1A with the other immune response related proteins RAR1 and SGT1, and with N-Rboh was also shown by YTH and suYTH. Finally, it was also demonstrated that the interaction of OsRACK1A with Rac1, RAR1, and SGT1 occurred on blades 1 and 2, indicating that the conserved region 1 was mediating this interaction. Subsequently, the same group also reported that the key transcription factor OsRap2.6 contributes to rice innate immunity through its interaction with RACK1A in compatible interactions (Wamaitha et al., 2012). These results established the role of plant RACK1 in innate immunity and resistance to rice blast fungus infection.

After the first rice report of RACK1-interacting ligands, Klopffleisch et al. (2011) reported an *Arabidopsis* signal-transduction interactome that included the heterotrimeric G protein subunits and the three *Arabidopsis* RACK1 isoforms. This was achieved by a high-throughput YTH system screening prey cDNA libraries from nine *Arabidopsis* tissues. After ten screens, seven ligands for AtRACK1A, four for AtRACK1B, and twenty four for AtRACK1C were identified (Table 1). Eight of these were confirmed by BiFC *in planta* and interestingly, novel unexpected functions were found. The nature of the identified AtRACK1 ligands revealed an involvement in cell wall formation and stress (Klopffleisch et al., 2011). Earlier work had suggested a more important function for AtRACK1A than AtRACK1B and AtRACK1C because AtRACK1A loss-of-function resulted in multiple defects in plant development while the loss of function of AtRACK1B and AtRACK1C did not (Guo and Chen, 2008). Therefore, it was unexpected that the largest proportion of ligands was obtained for AtRACK1C. Interestingly, no direct interaction among RACK1 isoforms and G protein subunits was found in this interactome (Klopffleisch et al., 2011). More plant RACK1 ligands were identified when AtRACK1A was used as a bait to systematically screen a suYTH-based *Arabidopsis* inflorescence cDNA library (Kundu et al., 2013). Ninety potential ligands were reported as involved in several functional plant processes such as oxidative, drought and saline stress, photosynthesis, and protein biosynthetic pathways (Kundu et al., 2013; Table 1). These data also demonstrated that the suYTH assay was more efficient than the YTH since more ligands were identified. It is likely that in this assay, the activation of the reporters was carried out in the cytoplasm where the interactors are found, and the probability to get positive interactions was higher. Kundu et al. (2013) also showed that phosphorylation on tyrosine 248 (Y248) of AtRACK1A was critical for the interactions since when a Y248F-RACK1A mutant was used in the suYTH assay, no positive interactions were observed. Importantly, this Y248 residue is conserved among *A. thaliana, H. sapiens, D. melanogaster*, and *S. cerevisiae*, and is located on blade 6 of the conserved region 2, at the bottom of the propeller (Ullah et al., 2008). These results also confirmed that tyrosine phosphorylation occurs on plant RACK1, and that it is required for both, homodimerization of AtRACK1A, and binding of interactors. Within the identified ligands in this report, four kinases were found: CLV1-like leucine-rich repeat transmembrane receptor-like protein kinase, AGC (cAMP-dependent, cGMP-dependent and protein kinase C family protein), PGK1 (Phosphoglycerate kinase 1), and WNK8 (WITH NO LYSINE (K) KINASE 8) protein kinase (see below). From these kinases, only WNK8 has been characterized as an atypical serine/threonine kinase that directly interacts with RACK1B and RACK1C, but not with RACK1A (Klopffleisch et al., 2011). Furthermore, YTH assays revealed that all three AtRACK1 proteins physically interacted with WNK8, and confirmation of this interaction was carried out on tobacco leaf epidermal cells by BiFC. Indeed, the three AtRACK1 isoforms were phosphorylated by WNK8 at threonine 162 and serine 122 (T162 and S122) without substrate specificity. The RACK1 residue S122 is also conserved in several species (*A. thaliana, O. sativa, H. sapiens, D. melanogaster*, and *S. cerevisiae*) and localizes on blade 3, while T162 residue is on blade 4 and is plant-specific. Blades 3 and 4 are in the conserved region 1, on the top rim of the propeller (Ullah et al., 2008). AtRACK1 was confirmed as a substrate of WNK8 and this phosphorylation negatively affected its stability. The phosphomimetic mutations on S122 and T162 abolished its expression at the protein level but the accumulation of the *AtRACK1* transcript was not affected. This suggested that AtRACK1 is controlled by phosphorylation and subsequent protein degradation (Urano et al., 2015). From these data, more information has been obtained regarding the binding properties of RACK1, new interacting ligands, and how phosphorylation affects this binding. However, the responsible tyrosine kinase critical for binding, and whether its association in a complex with AtRACK1 occurs, remain unknown and new strategies will have to be implemented to solve this enigma.

Growing evidences show that RACK1 is also involved in MAPK pathways. For example, in mammalian systems it has been reported that RACK1 induces cell proliferation via the MAPK cascade in association with the ERK, JNK and p38 families (López-Bergami et al., 2005; Vomastek et al., 2007). While significant knowledge of the RACK1-MAPK cascade relationship in mammalian and yeast systems over the past two decades has emerged, much less is known on how these pathways function in plants. This is further hampered by the facts that plant cells do not have *bona fide* G protein-coupled receptors (GPCRs), canonical PKC, or any of the RACK1, G protein and MAPK-linking chaperone orthologs of yeast *ste5* or mammalian beta arrestin (Witzel et al., 2012; Urano et al., 2013). Although, it was completely unknown how plant RACK1 contributed to the MAPK pathways, recently, Cheng et al. (2015) provided the first intriguing evidence that *Arabidopsis* RACK1 scaffolds the MAPK pathway through binding to the Gβ subunit. This finding links RACK1 to upstream G-protein signaling and downstream activation of a MAPK cascade in a pathogen-secreted, protease-mediated immune signaling pathway. They hypothesized that the 25% amino-acid sequence identity and similar seven-bladed β-propeller structure between RACK1 and Gβ might play a role in linking the G protein complex to downstream MAPK pathways. Their study showed that *Arabidopsis* RACK1 forms a complex with Gβ, MEKK1 (a MAPKKK), MKK4/MKK5 (two redundant MAPKKs), and MPK3/MPK6 in the presence of a pathogen-secreted protease treatment. Furthermore, they found that all three RACK1 protein isoforms bind the Gβ subunit. MPK3/MPK6 induced immune responses against pathogen infection through phosphorylation activation of their substrates. In a separate study in which RACK1 association was not investigated, Lieberherr et al. (2005) showed that attenuation of *OsRac1* by RNAi-mediated knockdown or loss of function of Gα (termed as *dwarf1*), drastically decreased OsMAPK6 levels. They found that OsMAPK6 and active but not inactive OsRac1 formed a protein complex in rice immunity. It is worth noting that Ras-MAPK or G protein-MAPK cascades occur in response to various stimuli, such as hormones or environmental stresses (Kawano et al., 2010). Since OsMAPK6 and OsRac1 proteins are in the same protein complex in the immune signaling pathway, it can be envisaged that OsRACK1 might have a functional link in this complex. However, unlike *Arabidopsis*, a direct evidence showing that OsRACK1 scaffolds the rice immunity signaling pathway with any member of the MAPK superfamily has not been reported so far and remains to be elucidated.

The heterotrimeric G protein is composed of three different canonical subunits (α, β, and γ), which in *Arabidopsis* are named GPA1, AGB1, and AGG1/AGG2 (there are two genes encoding the Gγ subunit in *Arabidopsis*). Before 2011, all the screens identifying one of the heterotrimeric G protein subunits interacting with RACK1 had been in animals. However, the essential residues for this interaction were already identified on blades 1–4 of plant RACK1 and thus, the conserved region 1 could mediate binding to the Gβγ dimer and the Gα*βγ* trimer (Dell et al., 2002; Ullah et al., 2008). Earlier reports documented that AtRACK1A, AtRACK1B, and AtRACK1C did not interact directly with AGB1 as tested by YTH, suYTH, and Co-IP assays (Guo et al., 2009b). However, a recent study demonstrated that all three AtRACK1 isoforms interacted with AGB1 by three methods (Cheng et al., 2015). First, BiFC in *Nicotiana benthamiana* leaves demonstrated that RACK1A, RACK1B, and RACK1C interacted with AGB1, MEKK1(K361M), MKK4, MKK5, MPK3, and MPK6, but not GPA1 or MPK4. Second, the interactions were further confirmed only for AtRACK1A by means of BiFC and split firefly luciferase complementation (SFLC) assays in *Arabidopsis* protoplasts. Third, binding between all three *Arabidopsis* RACK1 proteins and AGB1 was observed in co-immunoprecipitation experiments with *Arabidopsis* mesophyll protoplasts using Flag-tagged RACK1 proteins as the bait and HA-tagged AGB1 as the prey. In contrast, no binding was observed when HA-tagged GPA1 was used as prey (Cheng et al., 2015). This further supported the central role of RACK1 in innate immunity, as previously shown for *O. sativa* (Nakashima et al., 2008). In addition, Olejnik et al. (2011) later reported that AtRACK1A bound to AGG1, AGG2, and AtNUDT7. The latter is a pyrophosphatase capable of hydrolyzing NADH and ADP-ribose *in vitro*, and plays a role in the response to biotic and abiotic stresses. These data suggested a correlation between heterotrimeric G protein function and stress responses.

The data indicates that GPA1does not directly interact with RACK1 in any of the implemented assays (YTH, suYTH, Co-IP, *in planta* BiFC, SFLC, etc.). Moreover, although the interaction between RACK1 and the AGG1/AGG2 subunits was not analyzed in these reports, it was previously shown that AtRACK1A consistently interacted with both AGG1 and AGG2 using *in vitro* (pull-down), and *in vivo* (YTH and BiFC in protoplasts) assays (Olejnik et al., 2011). Therefore, the accumulating evidence leads to the notion that plant RACK1 can form complexes with the Gβ and Gγ subunits, but not with Gα. Thus, it is likely to participate in the regulation of some heterotrimeric G protein functions such as pathogen-activated immune response and oxidative stress, which are processes where other plant RACK1 interactors have been identified. Nevertheless, plant RACK1 participation in G protein-mediated developmental processes as found in other models (Omosigho et al., 2014), should not be ruled out.

## Concluding remarks and future directions

It is clear that plant RACK1, in analogous manner to its animal homolog, has earned itself a name in the WD-repeat protein family as a promiscuous but multi-interactive protein key for many critical plant processes and responses.

Plant RACK1 ligands remained elusive for almost two decades after the report of the first homolog in tobacco; however due the growing interest in RACK1 function, and the development of new analytical techniques, a total of 138 ligands have been identified so far (Table 1). This identification allowed to assign RACK1 function to a wide range of biological processes in which RACK1 is the central scaffolding molecule.

The particular feature of RACK1 to scaffold multiprotein complexes, also render it with the capability to participate in a wide variety of cell processes. Its properties result in the spatio-temporal regulation of critical signal-transduction events in plants including hormonal control, stress responses, development, immune defense, protein translation regulation, miRNA production, photosynthesis, and cell wall biogenesis. A central participation in all these processes require its constitutive and ubiquitous expression in order to carry out its varied and exquisite functions.

Although, significant progress has been made, the mechanisms that control its expression and functions are not yet well understood. It is now known that its expression is, in part, affected in a species- and tissue-specific dependent fashion by hormones (auxins, gibberellins, cytokinin, etylene, ABA, brassinosteroids, and methyl jasmonate), phosphorylation (Tyr248, S122, and T162), stress, and some ligands. An example of the species-specific differential regulation of RACK1 in plants is the distinct marked effect produced by ABA, which functions as a negative regulator of *RACK1* expression in *Arabidopsis*, but does not have any effect on the expression of *arcA* in *N. tabacum*. On the other hand, it induces the expression of *RACK1* in *O. sativa, M. truncatula, P. vulgaris*, and *Z. mays*. It is important to point out that the variation of the plant RACK1 expression patterns in different species could be the result of tissue-specific regulation. For example, these assays were carried out in different tissues and organs such as seedlings (*A. thaliana*), roots (*M. truncatula* and *P. vulgaris*), leaves (*Z. mays*), and cultured cells (*N. tabacum* and *O. sativa*). Furthermore, a differential regulation of RACK1 expression induced by auxin was observed between germination (slight down-regulation) and root development (up-regulation) in the same plant, *P. vulgaris*. In addition, it was shown recently that RACK1 becomes unstable and undergoes protein degradation upon phosphorylation by WNK8 on serine 122 and threonine 162 residues. Furthermore, RACK1 expression is also controlled by some ligands such as Rac1, which associates in an immune complex, thereby regulating its expression at transcriptional and post-transcriptional levels. RACK1, in turn, regulates Rac1 transcription in a feedback loop. In summary, RACK1 regulation in plants is carried out at several levels which involve responses to hormones, post-translational modifications such as phophorylation, and direct interactions with ligands.

Another salient feature of its functional versatility is that RACK1 forms a structural part of the 40S ribosome and thus, it is involved in regulation of protein expression. This is substantiated by the facts that: (a) several ribosomal proteins were identified as ligands (Figure 1; Table 1); (b) it binds to the translation initiation factor eIF6 A and B to maintain the 60S ribosome biogenesis and whose phosphorylation leads to 80S monosome assembly; and (c) it participates in the pre-miRNA processing via interaction with the SE protein, increasing the processivity and transcription/stability of certain pri-miRNAs.

Finally, defense against pathogens has emerged as one of the main processes in which RACK1 is involved in plants (Table 1). As shown for *O. sativa* and *Z. mays*, it is involved in the formation of the immune complex that participates in the resistance against the rice blast fungus, where it directly interacts with Rac1, RAR1, SGT1, and Rboh. RACK1 also interacts directly with the pyrophosphatase NUDT7, which is induced by bacterial pathogens and abiotic stressors. In addition, the relationship between RACK1 and processes of stress, photosynthesis and cell wall biogenesis became apparent since a dozen, eighteen and seven interactors, respectively, were indentified for each process (Table 1). These are novel processes in which RACK1 had not been implicated until recently. These recent findings highlight the importance to study and identify new interactors and their mechanisms that regulate and dictate when and where RACK1 will bind to channel its participation in a particular process.

Even though *A. thaliana* has been used traditionally as a plant model and copious research on RACK1 has been carried out on this species, future studies will likely move into the direction of searching for RACK1 functions in plant systems with smaller RACK1 gene families and/or in which one isogene predominantly expresses such as *P*. *vulgaris, O*. *sativa*, or *Z*. *mays*.

In conclusion, significant light has been shed regarding the role of plant RACK1 function in critical plant physiological pathways so far (Figure 2). With the ever flowing advent of new technologies and the availability of simpler plant models, the fine dissection of the interaction mechanistics and the unequivocal identification of critical RACK1 interactors should be achieved in order to unveil the whole of plant RACK1 function.

## Author contributions

TI wrote part of hormonal signaling, interacting ligands, part of concluding remarks, assembled table and drew Figure 1, provided critical comments and reviewed all the manuscript. AR wrote part of miRNA, drew Figure 2, HU wrote part of miRNA, protein translation, drew Figure 2, provided images, provided critical comments and reviewed all the manuscript. MV wrote introduction, part of hormonal signaling, immunity, stress, part of concluding remarks, reviewed, and coordinated writing of the manuscript.

### Conflict of interest statement

The authors declare that the research was conducted in the absence of any commercial or financial relationships that could be construed as a potential conflict of interest.
